# A High-Precision Temperature Control System for Electrochemical Measurements of Lithium-Ion Batteries and Individual Components

**DOI:** 10.1021/acsomega.6c00893

**Published:** 2026-05-27

**Authors:** Robson L. Grosso, Shirley L. Reis, Uesley A. Stival, Ira B. C. Gallo, Adler Souza, Juliane B. Kosctiuk, Francisca E. R. Oliveira, Beatriz Leao, Marianne G. S. Franchetti, Cyrille F. N. Gonin, Heverson R. Freitas, Marcos A. C. Berton

**Affiliations:** SENAI Institute for Innovation in Electrochemistry, Curitiba, Paraná 82590-300, Brazil

## Abstract

Temperature is critically important for lithium-ion batteries (LIBs) because it significantly impacts their performance, lifetime, and safety. Thermal control plays an important role in the reliability and safety of LIBs, since extreme temperatures can drastically reduce battery capacity and efficiency, implying irreversible damage, such as side reactions, leakages, and explosions. Consequently, a thorough understanding of the electrochemical behavior of the battery and individual components as a function of temperature is crucial for application. In this work, a temperature control system was designed using Swagelok-type cells and a silicone rubber heater, enabling a systematic electrochemical characterization across a wide temperature range up to 250 °C with high precision. Quantitative validation of the proposed system demonstrates significantly improved thermal stability compared to a conventional box furnace due to the localized heating configuration. At set temperatures of 40 and 70 °C, the developed system maintained stable profiles of 42 ± 0.4 and 71.5 ± 0.6 °C, respectively. To demonstrate its applicability, NMC811 half-cells were evaluated at 25 and 45 °C. Furthermore, the ionic conductivity of thin flexible polymer and dense ceramic solid electrolytes was determined within temperature ranges of 25–70 °C and 25–250 °C using electrochemical impedance spectroscopy to demonstrate the versatility of the temperature control system on evaluation of both battery cells and individual components. The results confirm that the proposed setup enables reliable electrochemical characterization with high temperature stability.

## Graphical abstract

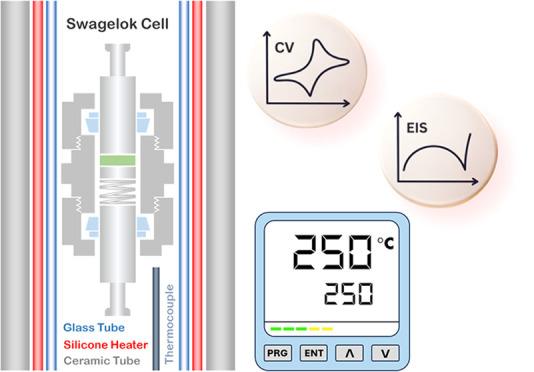

## Introduction

Lithium-ion batteries (LIBs) are essential to modern energy storage systems, powering a wide range of applications from portable electronics to electric vehicles and grid-scale energy storage systems.^1,2^ Their high energy density, long cycle life stability, and relatively low self-discharge characteristics make LIBs highly suitable for such demanding technological needs. Moreover, ongoing advancements in materials science and battery technology continue to improve their performance, safety, and cost-effectiveness, further consolidating their role in the energetic transition toward sustainable and renewable energy solutions.^1,2^ Temperature plays an important role in LIBs because it significantly impacts their performance, lifetime, and safety. In this context, understanding of the thermal behavior of LIBs and their individual components (i.e., electrodes, electrolytes, and separators) is crucial for safety and reliability, since extreme temperatures can drastically reduce battery capacity and efficiency, potentially implying irreversible damage such as side reactions, leakages, thermal runaway, and explosions during their usage.^1^

Electrochemical measurements, such as cyclic voltammetry (CV), electrochemical impedance spectroscopy (EIS), and galvanostatic charge/discharge (GCD) cycling, are widely used to evaluate the behavior and degradation of LIBs and their individual components.^2–4^ However, these measurements can be significantly affected by temperature fluctuations, which introduce inconsistencies and reduce the reliability of the experimental data. Although there are several commercial furnaces available, loss of heating due to high-volume chambers, for example, makes precise temperature control difficult. Moreover, thermocouples in tube furnaces are often designed to measure temperature outside the tube, promoting inconsistencies with actual sample temperature. The importance of temperature control has been recently discussed for the lithiation process of LiNiO_2_ (LNO),^5^ which is a high-capacity cathode material used in LIBs. The authors found a wide range of temperatures from 550 to 750 °C among 34 reports published in the literature. Furthermore, the lithiation reaction using Ni­(OH)_2_ and LiOH to produce LNO was performed in three different high-temperature furnaces for precise temperature control evaluation. All furnaces showed a significant variation of about 40 °C in the steady temperature compared to the value indicated on the furnace controller, and overshoot close to 10 °C to the maximum set temperature.^5^ It has been demonstrated that these temperature fluctuations induced significant changes in the battery capacity and rate performance behavior of LNO.^5^

Thus, it becomes evident that a deep understanding of the electrochemical properties of the battery and its individual components as a function of temperature is critical for application. To address accurate, reproducible, and meaningful electrochemical characterization, this work focuses on the development of an affordable temperature control system tailored for electrochemical measurements of LIBs and their components, aiming to enhance high-precision measurements. For this purpose, stainless steel (SS) Swagelok (SWK) cells have been used.

SWK fitting is a gas tube connector originally designed to create a leak-tight connection between tubing. Slight modifications in this connector allow for usage as a powerful tool for electrochemical measurements. As a battery cell type, SWK cells are well-known for being reusable, easy to assemble and disassemble, and versatile.^6–11^ This cell format has been used for electrochemical characterization of batteries and supercapacitors since the 1980s.^6,7^ The versatility of SWK cells has been demonstrated in the literature, where different modifications allow a broad range of investigations. In pioneer studies, a PTFE polymer SWK cell with SS plungers was used to design an experimental methodology to quantify sources of variation in battery testing procedures for lithium/fluorinated carbon (CF_*x*_) systems.^6^ SWK cells can be set up with both two and three probes. While the former is usually used in a standard coin-cell configuration with anode as counter and reference electrodes (CE and RE, respectively) and cathode as the working electrode (WE), both electrodes are sandwiched with a separator in between. The latter has a T-shape design and is used as an *in situ* diagnostic tool by inserting the third electrode, which is the RE, allowing quantitative evaluation of distinct contribution of each cell component to the overall battery performance.^12–14^

Recently, by inserting a pressure sensor device in one of the metal plungers, a two-probe cell configuration was designed for operando pressure measurements.^11^ The authors demonstrated that a modified SWK cell enables high-sensitivity detection of small amounts of gases and electrode volume changes during electrochemical cycling for LiFePO_4_ (LFP) and graphite electrodes in half-cell configurations. Electrochemical measurements as a function of temperature are often carried out in laboratory-scale box furnaces, allowing temperature control during the testing. However, thermal fluctuations are subject to occur due to the large internal chamber of the box furnace compared to the small SWK size. To address this point, an interesting approach has been applied for high-temperature electrochemical measurements in LIBs. A two-probe SS SWK cell was designed and optimized for testing in a wide temperature range from room temperature up to 300 °C.^9^ The authors modified one of the plungers and inserted an electrical insulator alumina ceramic tube to test LFP half-cells with lithium bis­(trifluoromethanesulfonyl)­imide (LiTFSI) as the molten salt electrolyte at 250 °C. However, reactions between the electrolyte and the ceramic plunger were found to be critical for this system. Moreover, it is susceptible to fractures, which might occur due to the brittle characteristic of ceramic materials,^9^ being even more critical for long-term measurements, such as GCD and rate capability (RC) testing.

Although SWK cells have shown versatility and reliability for electrochemical evaluation of batteries and supercapacitors, due to the popularity of coin-cell battery components and assembly machines, this powerful electrochemical-type cell has been poorly explored. In the current work, a high-precision temperature control system has been designed with low-cost materials to allow a broad range of electrochemical characterizations as a function of temperature, such as open circuit voltage (OCV), CV, GCD, RC, EIS, and self-discharge rate, among others.

## Results and Discussion

Representative images and diagrams of the temperature control system are presented in Figure 1. The system consists of a digital PID (proportional-integral-derivative) controller, connected to a solid-state relay, *K*-type thermocouple, and a silicone rubber heating element wrapped in a borosilicate glass tube. High-temperature isolation tape was used to fix the heating element on the external glass tube wall. A photograph of the system is shown in Figure 1a. An electrical diagram showing the details of the connections is presented in Figure 1b. Electrochemical measurements of battery cell assembly as well as individual battery components are carried out using a 316 SS SWK cell (Figure 1c,d). In this cell, PFA polymer double ferrule fittings are used to avoid short-circuit electrical and ensure leak-proof sealing. The electrical insulator nature of PFA prevents contact between plungers and the SWK union body. Furthermore, ferrules create a physical barrier that prevents the leaking of liquid electrolytes, as well as contact with O_2_ and moisture from the atmosphere. Electrochemical analyzers can be connected to the SWK cell via cables using a plier-like spring-tensioned metal clip (“alligator clip”) or pin connectors plugged directly to the holes on top of SWK plungers. For the electrochemical measurements, the SWK cell assembly is placed inside the glass tube, and the entire system is set inside the ceramic tube to prevent thermal fluctuations. In this sense, ceramic fiber blankets can be placed on both sides of the ceramic tube to ensure temperature isolation.

**Figure 1:**
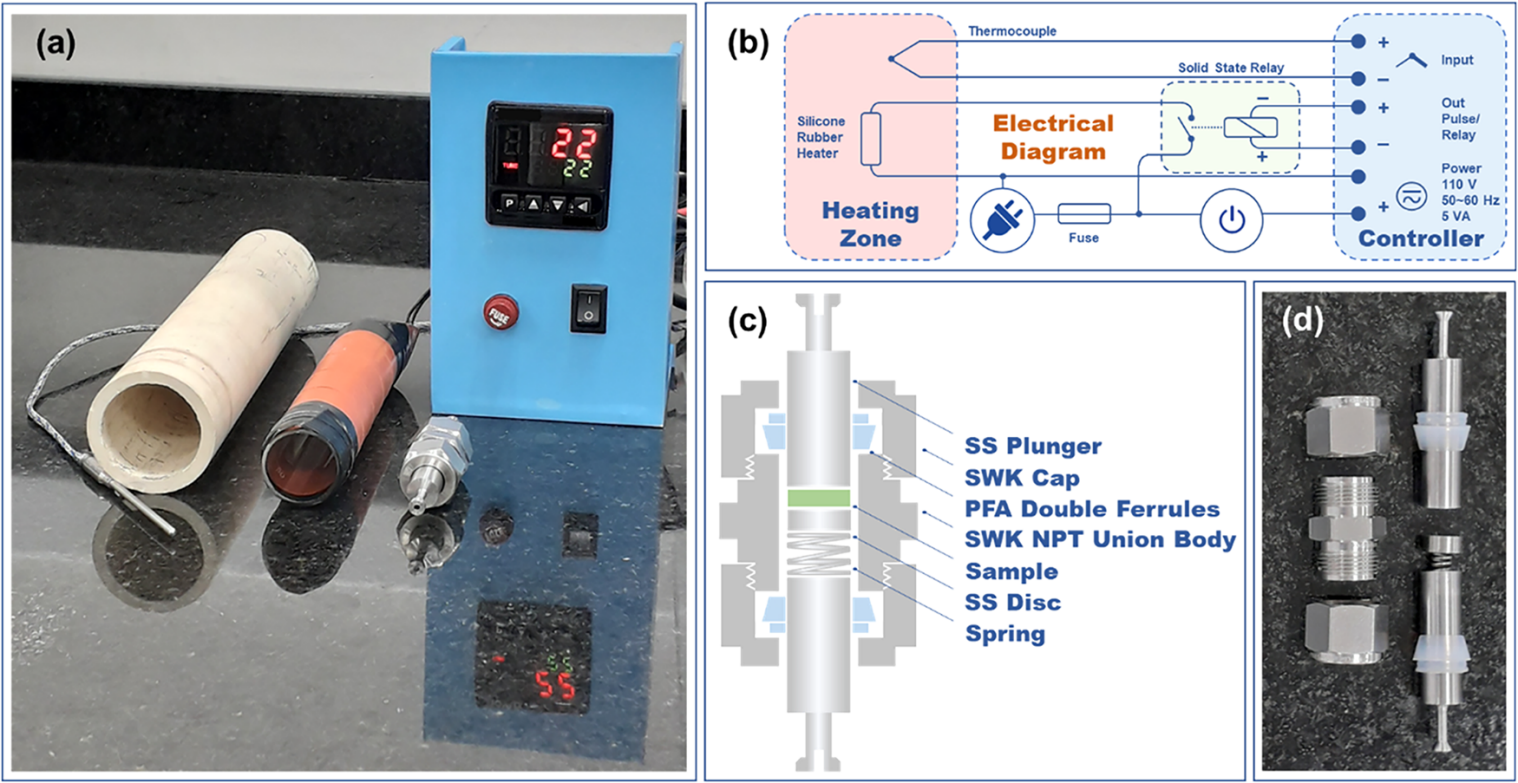
Temperature control system for electrochemical evaluation of batteries and their components. (a) Photograph of the disassembled system, (b) electrical circuit diagram, (c) schematic representation of the Swagelok (SWK) cell, and (d) photograph of the SWK cell.

Silicone rubber heaters are thin, flexible, lightweight, chemical resistant, and flame retardant and can reach temperatures up to ≈250 °C.^15^ This heating element consists of a high-temperature resistance wire wound vulcanized between two sheets of silicone rubber. The silicone sheets have a thin fiberglass layer to allow for the strength and tear resistance of the heater. Furthermore, borosilicate glass is a well-known labware and shows compatibility with silicone rubber due to its low thermal expansion coefficient of ≈3 × 10^–6^ K^–1^ at 20 °C and thermal stability up to ≈400–500 °C.^16^ The thermal conductivity of borosilicate glass, which is ≈1 W m^–1^K^–1^, allows for a quick heat transfer from the heating element to the sample via conduction and convection mechanisms. In addition, 316 SS shows a thermal conductivity of ≈16 W m^–1^K^–1^ and a relatively low thermal expansion coefficient of ≈16 × 10^–6^ K^–1^ between 20 and 200 °C. Thus, it becomes evident that the thermal properties of the different materials used to design the temperature control system are very similar, which is desired under testing conditions.

Unlike the previous reported high-temperature SWK cell, where direct ceramic–metal contact may introduce stress-induced fracture and electrolyte–ceramic reactions,^9^ the present design incorporates a glass barrier and an external silicone heater, effectively decoupling thermal control from mechanical compression. This configuration mitigates chemical instability and improves structural durability. The use of the silicone rubber heater as an external heating element allows uniform radial heating, reducing thermal gradients that contribute to mechanical stress and brittle fracture.

Quantitative validation of the temperature control system is demonstrated in Figure 2, where the temperature evolution was monitored as a function of time within 60 min, which is a typical interval employed for EIS measurements, for example. Temperature measurements were compared with data collected in a conventional box furnace at two representative set points of 40 and 70 °C, as shown in Figure 2a and 2b, respectively. As evidenced for both set points, the conventional box furnace exhibits significant overshoot and fluctuations. At 40 °C (Figure 2a), the temperature oscillates from 39 to 51 °C, which corresponds to a variation of 12 °C. Fluctuation at the 70 °C set point (Figure 2b) was evidenced from 69 to 80 °C with a delta of 11 °C. On the other hand, in the designed system, oscillations at set points of 40 and 70 °C were found to be 41.6 to 42.3 °C (42 ± 0.4 °C) and 70.9 to 71.9 °C (71.5 ± 0.6 °C), respectively, corresponding to a delta <1 °C. Although the average absolute deviation ranges from 2 to 1.5 °C above the set point at 40 and 70 °C, respectively, the proposed temperature control system rapidly reaches a stable temperature with minimal overshoot and maintains with small amplitude in the temperature profile throughout the measurement period. The improved stability can be attributed to the localized heating configuration implemented in the designed system. Thus, the quantitative validation analysis demonstrates that the developed setup provides improved temperature stability compared to conventional furnace heating, enabling reliable electrochemical measurements where precise thermal control is highly required.

**Figure 2:**
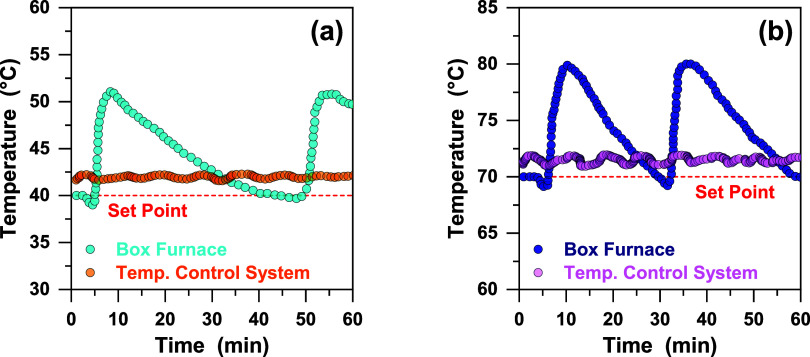
Temperature stability profiles measured as a function of time for a conventional box furnace and the developed high-precise temperature control system at set points of (a) 40 °C and (b) 70 °C. The dashed red line indicates the set temperature.

The electrochemical performance of half-cell LIBs with the NMC811 cathode as a function of temperature was evaluated, aiming to demonstrate the applicability of the developed system. Results obtained for half-cells are presented in Figure 3. To verify the effect of temperature during cycle life, all cells evaluated were submitted to formation cycles at 25 °C using a *C*-rate of 0.1C. As an example of a typical result, the second GCD cycle of formation for one of the evaluated cells is shown in Figure 3a. Discharge specific capacity values of around 200 mAh g^–1^ with a Coulombic efficiency of >99% were obtained during formation. The effect of temperature on battery performance was evaluated by using a fast-charging *C*-rate of 2C within 100 cycles (Figure 3b) at 25 and 45 °C. As can be observed, discharge capacity is higher in the initial cycle at 45 °C. An improvement of ≈10% from 161 to 178 mAh g^–1^ at 2C was obtained by increasing the cell temperature to 20 °C. However, it becomes evident that capacity declines faster at higher temperatures, where capacity retention values were found to be 71% at 25 °C and 68% at 45 °C with an ≈5% difference in capacity in the 100th cycle.

**Figure 3:**
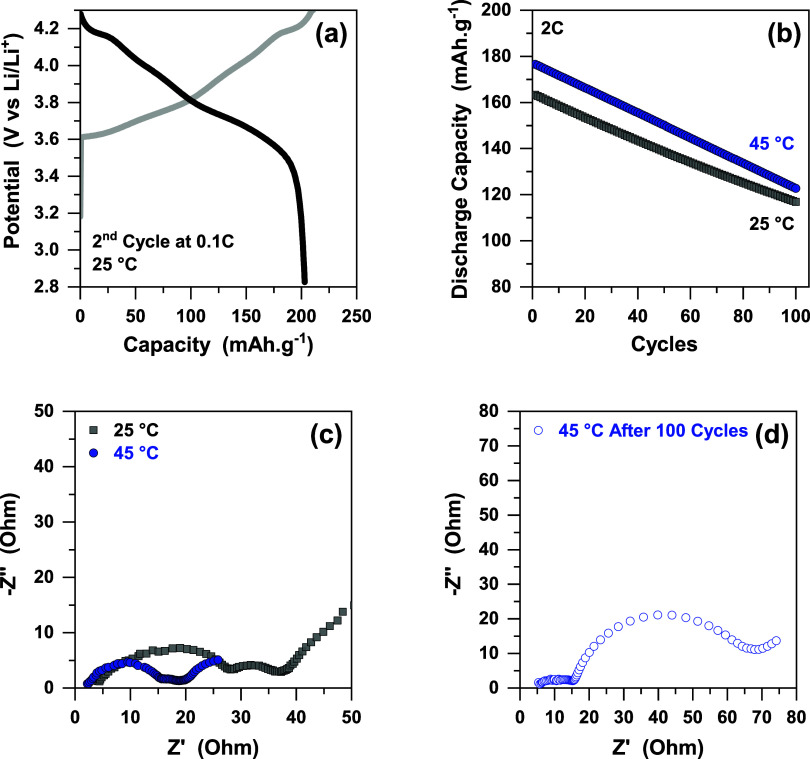
Electrochemical data of NMC811 half-cell lithium-ion batteries. (a) Charge/discharge curves obtained during the 2nd cycle of formation using a *C*-rate of 0.1C at 25 °C, (b) cycle life performance at 2C for two different temperatures (25 and 45 °C), and impedance diagrams (c) obtained after formation followed by stabilization at 25 and 45 °C, and (d) after 100 GCD cycles at 45 °C.

EIS measurements were performed to evaluate charge-transfer resistances (*R*_ct_) of the cells at different temperatures before cycling (Figure 3c) and after cycling at 40 °C (Figure 3d). Impedance diagrams obtained exhibit typical features observed in LIBs: initial resistance (*R*_i_) followed by two semicircles corresponding to the electrolyte/electrode interface resistance (*R*_s_) at high frequencies and *R*_ct_ at the intermediate-frequency range. More information can be found in ref 17. A significant decrease in *R*_ct_ is observed with an increasing temperature, dropping from 8.5 Ohm at 25 °C to 3 Ohm at 45 °C.

Although an increase in temperature decreases the *R*_ct_ of the cell before GCD cycling, results indicated that the degradation process is accelerated by increasing temperature.

This behavior might be attributed to side reactions accelerated at elevated temperatures, such as oxidation–reduction reactions of the liquid electrolyte,^18^ SEI decomposition or uncontrollably growth,^19^ and transition metal dissolution, which has been reported for NMC causing migration of ions to the anode, damaging SEI and further reducing capacity.^20^ These side reactions consume active Li and electrolyte, leading to capacity fading and increased impedance, as can be observed in the impedance diagrams at 40 °C in Figure 3c and 3d, where a significant increase in *R*_ct_ is observed from 3 Ohm before cycling (Figure 3c) to 50 Ohm after 100 cycles (Figure 3d). Thus, high temperature makes degradation reactions thermodynamically and kinetically more favorable. Although moderate heating can improve performance in the short term, sustained elevated temperatures accelerate capacity fade, shorten cycle life, and increase safety risks.

It is important to note that all electrochemical measurements were performed using independently assembled cells under identical conditions, confirming the reliability of the observed temperature-dependent trends. The NMC811 half-cell was employed as a model system to validate the performance of the developed temperature control system. Furthermore, impedance diagrams presented in this work represent the possibilities of in-depth electrochemical analysis that the proposed highly precise temperature control system might allow.

Battery components were also evaluated to demonstrate the versatility of the temperature control system. Ionic conductivity values were determined from 25 to 70 °C for the poly­(ethylene oxide) (PEO) polymer^21^ and from 25 to 250 °C for the Li_5_La_3_Nb_2_O_12_ (LLN) garnet-structured ceramic.^22^ Experimental data are presented in Figure 4. As evidenced by Arrhenius plots shown in Figure 4a and 4b for PEO and LLN, respectively, ionic conductivity in both solid electrolyte materials increases further with temperature, with a slope shift indicating a change in conduction mechanisms. PEO and LLN show ionic conductivity values of 1.28 × 10^–5^ and 1.93 × 10^–5^ S cm^–1^, respectively, at 25 °C. Similar values were reported in the literature for both solid electrolytes.^21–24^ The change in slope observed in the Arrhenius plot for PEO (Figure 4a) is consistent with the crystalline-to-amorphous transition, as reported in previous studies.^21,23,24^ In addition, differential scanning calorimetric (DSC) studies confirmed phase transition between 50 and 60 °C for PEO-based electrolytes.^21,23,24^ This transition leads to enhanced segmental mobility and a reduction in activation energy for ion transport from 0.88 eV (25 to 50 °C) to 0.38 eV (50 to 70 °C). Similarly, a change in the slope in the Arrhenius plot for LLN is observed at 125 °C (Figure 4b) with activation energies of 0.51 eV from 25 to 125 °C and 0.28 eV from 125 to 250 °C. This phase transition has been reported for garnet-type solid electrolytes, associated with changes in Li^+^ migration pathways within the crystal structure at elevated temperatures.^25^ Representative impedance diagrams obtained for LLN at 40 and 60 °C are shown in Figure 4c to demonstrate an increase in ionic conductivity as a function of temperature. Both diagrams were fitted by a semicircle to calculate ionic conductivity, as described in detail in ref 22.

**Figure 4:**
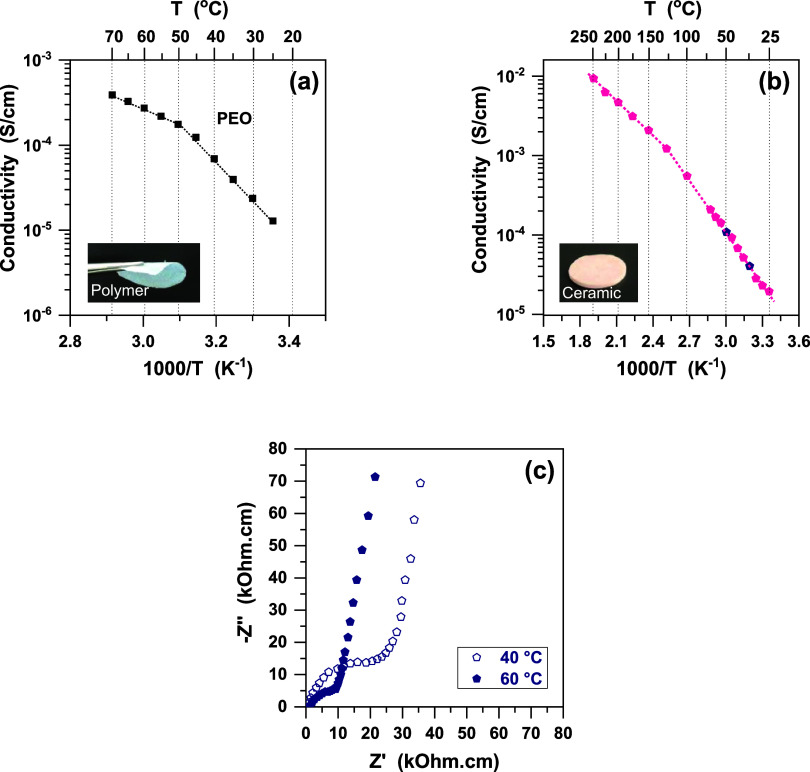
Experimental electrochemical data for battery components. Arrhenius plot of the ionic conductivity of the (a) thin, flexible PEO polymer and (b) LLN sintered dense ceramic. (c) Impedance diagrams of LLN at 40 and 60 °C.

## Conclusions

A highly precise temperature control system based on SWK-type cells using a silicone rubber heater was developed and validated for electrochemical characterization of LIBs and individual components. The system demonstrated excellent thermal stability with temperature fluctuations below 1 °C, significantly outperforming a conventional box furnace, which exhibited oscillations above 10 °C. The applicability of the designed system was demonstrated through electrochemical evaluation of NMC811 half-cells at 25 and 45 °C. While moderate heating improved the initial discharge capacity with reduced *R*_ct_, accelerated degradation was observed during GCD cycling tests at 45 °C. The experimental results highlight the importance of precise thermal control for reliable battery testing.

The versatility of the system was further demonstrated through EIS measurements of polymer and ceramic solid electrolytes within a broad range of temperatures up to 250 °C, enabling accurate ionic conductivity evaluation. Overall, the proposed system provides a simple, low-cost, and reliable platform for studying temperature-dependent electrochemical processes in energy storage materials. Thus, the system can be readily applied to studies of battery degradation mechanisms, accelerated aging experiments, and characterization of advanced solid electrolytes, contributing to an improved understanding and development of next-generation energy storage technologies.

## Methods

### Temperature Stability and Control Accuracy

Temperature stability measurements were performed to evaluate the accuracy and thermal response of the developed temperature control system. The temperature was monitored as a function of time by using a calibrated *J*-type thermocouple (Omega, TJ72-CPSS-116U-6) positioned at the same location where the sample under evaluation is typically placed. Two representative temperature set points, 40 and 70 °C, were selected. The temperature was recorded continuously for 60 min. For comparison purposes, the same procedure was performed in a high-temperature box furnace (Jung, LF0912) with internal dimensions in cm of 16 × 16 × 35 (height × length × width).

### Half-Cell Batteries

The cathode electrode was produced using Li­(Ni_0.8_Mn_0.1_Co_0.1_)­O_2_ (NMC811) (MSE Supplies) as the active material, LITX (Cabot Corporation) as conductive carbon, and poly­(vinylidene fluoride) (PVDF) as the binder, in a weight proportion of 90:5:5. A slurry containing 50% (w/w) of powders in *N*-methylpyrrolidone (NMP) was prepared under constant stirring and deposited on aluminum foil (MSE Supplies) via tape casting. Half-cells were assembled into an argon-filled glovebox (MBraun, UNIlab Pro SP) using 10 mm diameter cathode electrodes with 4 mg cm^–2^ mass loading, lithium metal disks (99.9%, Alfa Aesar) as anode and reference electrodes simultaneously, and 12 mm diameter polypropylene (PP) sheets (Nanografi) as separators. 80 μL of 1 M LiPF_6_ in EC:DMC (1:1 v/v) (Sigma-Aldrich) liquid electrolyte was added to each cell evaluated.^17^

Electrochemical measurements were carried out using a Parstat potentiostat 4000A within a voltage window of 2.8–4.3 V vs Li/Li^+^ and 1C = 200 mA g^–1^ as a reference. Three cycles of formation at constant-current and constant-voltage (CCCV) at 0.1C with a 0.025C (C/40) cutoff were performed at 25 °C before heating testing. GCD experiments were evaluated using a *C*-rate of 2C for charge and discharge during 100 cycles at 25 and 45 °C. EIS (Solartron, SI 1260) measurements were performed within a frequency range of 1 Hz–10 MHz with an amplitude of 10 mV after formation cycles, followed by 1 h stabilization into the temperature control system at 25 and 45 °C. EIS was also performed after 100 cycles at 45 °C.

### Solid Electrolytes

Cubic garnet-type structured Li_5_La_3_Nb_2_O_12_ (LLN) powder was synthesized by a solid-state reaction method. Dense ceramic pellets were produced by spark plasma sintering (SPS, GT Advanced Technologies Inc.) at 950 °C for 10 min by applying a pressure of 50 MPa. Details of the LLN production and characterization were published elsewhere.^22^ A PEO polymer solid electrolyte was prepared inside a glovebox with O_2_ and moisture levels below 0.1 ppm. Initially, LiTFSI was slowly dissolved in acetonitrile with vigorous stirring at room temperature. After complete dissolution of the salt, PEO was slowly added to the solution, stirred for 3 h, drop-cast, and dried for 48 h. The ratio between PEO ethylene oxide units and Li^+^ ions (EO:Li^+^) used was 18:1. More information was previously reported.^21^ Au electrode layers were deposited on both sides of the solid electrolytes by the physical vapor deposition (PVD) technique (MBraun, EcoVap 5G) prior to the EIS measurements. Samples were assembled in a SWK cell and sealed. EIS was performed as a function of temperature from 25 to 70 °C for PEO samples and from 25 to 250 °C for LLN samples with 30 min of stabilization before measurement.

## †

Department of Nanoscience, Joint School of Nanoscience and Nanoengineering, University of North Carolina at Greensboro, Greensboro, North Carolina 27401, USA
